# Occupational burn injuries in Finland 2011–2015

**DOI:** 10.1186/s40621-022-00387-5

**Published:** 2022-08-26

**Authors:** Lotta Purola, Heli Kavola, Jyrki Vuola

**Affiliations:** 1 Department of Plastic Surgery, Oulu University Hospital, PO. Box 21, 90029 Oulu, Pohjois-Pohjanmaa Finland; 2 Department of Plastic Surgery, Helsinki Burn Centre, Helsinki University Hospital, HUCH, University of Helsinki, P.O. Box 800, 00029 Helsinki, Finland; 3 Hyvinkää Regional Hospital, Sairaalankatu 1, Hyvinkää, Finland

**Keywords:** Burn injury, Occupational, Work-related, Insurance, Return to work

## Abstract

**Background:**

This study comprises all hospitalized work-related burn injuries in one country during 2011–2015. The purpose was to describe demographics, causes and risk factors of occupational burn injuries with special focus on the outcome of return to work.

**Material and methods:**

This is a retrospective study on two data sources of which Finnish Workers’ Compensation Center’s (FWCC) register includes all work-related burn cases at a given time. Additional data have been obtained from those patients, who were referred to the National Burn Centre (NBC) during the same time according to the Emergency Management of Severe Burns (EMSB) criteria. We compare demographics, injury mechanisms and general burn data of these two patient groups.

**Results:**

Based on FWCC register, in 2011–2015 occurred 11,623 work-related burn cases of whom 54% were men. During the study period, NBC admitted 26 patients fulfilling EMSB criteria. The most severe patients treated in NBC had injuries affecting multiple body parts. In FWCC data, hand was most injured body part. Kitchen/bakery work was the most common profession in FWCC register but in NBC material industrial and transport professions dominated. In FWCC register, patients had lower mean age (37 years vs. 43 years). Most severe injuries occurred among older patients: In NBC data, those with total body surface area 40% or over had mean age 53 years. Majority of patients returned to work.

**Conclusion:**

Safety at work in Finland has improved during last decades, and the vast majority of work-related burn injuries are minor. Minor burn injuries are common in young adults working in kitchen and bakery work, whereas elderly men working in transports and industry sustain the most severe burn accidents. Retirement after work-related injury becomes very expensive for all parties, and this data can be used in preventing those cases as well as the minor accidents.

## Introduction

Burn injury is one of the most common traumas worldwide, and among adults, working-age men are the highest risk subgroup. Occupational burn injuries are common: in western countries, they comprise 15–30.2% of all medically treated burn injuries to working-age adults (Mian et al. 2011; Rossignol et al. 1986; Carroll et al. 1995; Ng et al. 1991). Their costs are higher than of those other occupational injuries, and 20% of the overall costs of burn care are due to absence from work (Haikonen et al. 2014; Hop et al. 2016; Onarheim et al. 2009; Smith et al. 2015).

Previous studies have found the total body surface area burned (TBSA) to be the most important predictor of overall recovery. The majority of occupational burn injuries are rather small, often hand injuries, with total TBSA less than 10%, enabling good recovery and return to work. A number of earlier studies have shown hand and/or upper arm injuries and older age having a negative association with the length of sick leave and return to work (Rossignol et al. 1986; Palmu et al. 2015; Goei et al. 2016). On the other hand, Mason et al. (2012) performed systematic review with 26 studies concerning return to work after burn injury. In their review, they reported that 70.03% of patients had returned to work after 41 months and that hand injuries often led to changes in job description. TBSA, surgical treatment and older age predicted poorer outcome (Mason et al. 2012).

Spronk et al. (2022) performed follow-up study among 213 adult burn patients utilizing burn-specific health-related quality of life (HRQL) questionnaire: 36% of patients had some form of activity impairment (Spronk et al. 2022). A number of factors concerning individual, health care units, workplace and social life have also been recognized to affect return to work (Oster et al. 2010).

Finland is a high-income country with a population of 5.5 million. Population density is low at 18.2 /m2, with a notable difference between urban and rural areas corresponding to geographical differences: Southern Finland is mainly urban with five major cities, whereas the northern part of the country is sparsely populated with large forest and wilderness areas. The cornerstone of Finnish welfare is education: The literacy rate is 99%, 66% of the population has a secondary education diploma, and 41% has a tertiary-level education. Nevertheless, the professions of men and women have traditionally differed in Finland (Immonen and Sutela 2021).

Taxation is the main funding of health and medical care in Finland. The public sector, including municipal health care and university hospitals, provides these services. The private sector also offers basic health care services, and many insurance companies work in cooperation with them.

As a part of the social security system, employers are legally obliged to insure their employees against occupational accidents and diseases. Private insurance companies entitled to practice health insurance business perform the functions of the law. Due to this obligation, occupational health services in Finland are of high quality and severe accidents are rare: In 50 years, the number of annual fatal accidents at work has fallen from over 90 to 30. Every employee has an obligation to report any, even minor, accident at work, and this is a prerequisite for claiming compensation: In the case of a work-related accident, the person is generally entitled to compensation for the loss of income, functional limitation, and rehabilitation. They are entitled to a workers’ compensation pension if they are unable to return to work after one year (Finlex 2015).

Since the 1960s, Finland has had a national civil register system of especial identification numbers. This enables a wide range of government-maintained national registers. The combination of these registers provides unique, even individual-level medical information. Different public organizations govern these, and data are available on the basis of detailed applications.

2015 employment rate in Finland was 68.1% (men 68.5%, women 67.7%) (Tilastokeskus 2015). Segregation of professions still exists in Finland: Three major sectors of employment covering 60% (1.3 million people) of overall professions have been service and selling (470,386 employees, women 72.8%), experts (423,292 employees, women 51.4%) and specialist experts (420,925 employees, women 58.3%). Firs two were also most common professions of women. Instead, most common profession of men were building, repairment and production work (228,900 men, 20.2% of employed men) (Pietiläinen 2013). Based on 2019 report by Statistics Finland, female majority professions were nursing and health care, teaching and experts of health care sector. Instead, male majority professions were science and technical experts, transportation and building (Tilastokeskus 2022).

In this study, we combined two data sources: the register data of the Finnish Workers’ Compensation Center (FWCC) and the patient data of the National Burn Centre (NBC). This approach enables the creation of unique data that cover all the work-related burn injuries in Finland in 2011–2015. To our knowledge, earlier studies utilizing both medical and insurance registers on occupational burn patients are either scarce or non-existent.

The aim of this study was to describe the demographics and risk factors of occupational burn injuries with a focus on the outcome of return to work. A second purpose was to provide new information for future preventive measures.

## Material and methods

### Data sources

In Finland, the municipalities form hospital districts, which provide second-level special health care at central hospitals (16 units). Five catchment areas organized around five university hospitals are responsible for highly specialized tertiary-level medical care. The treatment of some demanding, rare illnesses or diseases has been centralized nationally to one hospital or health care unit. All severe burns are treated in the NBC of Helsinki University Hospital.

The data were extracted from two sources:

- National Burn Centre (NBC)
  Records of occupational burn patients treated at the NBC in 2011–2015. Until 2016 there were two burn centers in Finland, one in Helsinki and another in Kuopio. Approximately 2/3 of burn patients requiring tertiary-level burn care were treated in Helsinki.
  Since 2016, the Helsinki Burn Centre has served the entire country as an NBC. Due to the local organization of medical care in the Helsinki and Uusimaa Hospital district, the NBC also treats minor burn injuries in the area.
  Helsinki University Hospital has an electrical patient information system which uses ICD-10 coding. In the case of any trauma, the report includes both the location and mechanism of the event. ICD-10 code Y96 represents occupational injury. Finnish legislation on occupational injuries is highly detailed, and due to this, these cases are reported promptly in health care.
  Our data included all occupational burn injuries from January 2011 to December 2015 treated at the NBC. Further analysis included occupational burn patients who met the Emergency Management of Severe Burns (EMSB) criteria of specialized burn care for adults (Breederveld et al. 2011). Referral criteria are based on Australian and New Zealand Burn Association (ANZBA) guidelines which cover patients with burns of ≥ 20% (10% for children and elderly) of the TBSA, ≥ 5% TBSA of full thickness burn, burns of special areas (i.e. , face, hands, feet, genitalia, perineum, major joints, circumferential limb/chest burns), burns with inhalation injury, electrical and chemical burns, burns with pre-existing illness or with associated trauma and burns on pregnant women. Patients who did not meet these criteria were excluded from further analysis.
- The Finnish Workers’ Compensation Center [Tapaturmavakuutuskeskus] (Finnish Workers' Compensation Center 2021)
  The FWCC collects information on compensated work-related injuries. All accidents that happen at the workplace must be reported according to Finnish legislation based on the European Statistics on Accidents at Work (ESAW) system (Eurostat 2013), which is different to the ICD-10 coding system.

The Information unit of the FWCC provided the data on compensated occupational burn injuries based on descriptive request from 2011–2015.

### Collected data

General demographics were collected from both data sources. The NBC provided medical information based on the ICD-10 coding system. The data contained the time of hospitalization, possible operations and outpatient visits. The FWCC data included injury type, injured body part, length of sick leave and total sum of compensations paid by insurance companies. It contained all reported cases, including those with no medical contact.

### Data handling

The NBC utilizes ICD-10 coding in its registers. In contrast, the FWCC utilizes ESAW coding. Due to this, data on professions, for example, were not directly comparable.

### Statistics

This study was epidemiological and descriptive in most purposes. Data analysis and visualization were performed using R software (Project R 2022). The differences between groups were presented by mean, median and standard deviations, according to adequacy. The Chi-squared test, Welch two-sample t test and ANOVA test were used for analyzing the differences between the groups as far as this was reasonable. The groups were assumed to have normal distribution.

The Research Ethics Committee of Helsinki University approved the study. None of the patients were contacted during the study.

## Results

Based on the FWCC register, between 2011 and 2015, 11,623 work-related burn injuries occurred in Finland (54% men). During the same time period, the NBC treated 26 occupational burn patients (96% men), who met the Emergency Management of Severe Burns (EMSB) referral criteria for specialized burn care. The NBC data had only one female (1/26), and the patients were older than those in the FWCC register, *p* value 0.004 (Table 1).

**Table 1:** Occupational burn injuries in Finland by age and gender, 2011–2015

Age	Male		Female		All	
	FWCC*	NBC**	FWCC	NBC	FWCC	NBC
< 18	103 (2%)	0	104 (2%)	0	207 (2%)	0
18–30	2354 (38%)	8 (31%)	2155 (41%)	1 (100%)	4509 (39%)	9 (35%)
31–40	1429 (23%)	0	863 (17%)	0	2292 (20%)	0
41–50	1212 (19%)	8 (31%)	968 (19%)	0	2180 (19%)	8 (31%)
51–60	918 (15%)	6 (24%)	927 (18%)	0	1845 (16%)	6 (23%)
61–70	206 (3%)	3 (12%)	186 (4%)	0	392 (3%)	3 (12%)
71-	3 (0.05%)	0	1 (0.02%)	0	4 (0.03%)	0
Total	6225 (54%***)	25 (96%***)	5204 (46%***)	1 (100%***)	11,492	26
Mean *age*	37	43	36	28	37	43
Median *age*	35	47	34	28	35	47
SD	13	14	14		13	14

^*^Finnish Workers’ Compensation Center

^**^ National Burn Center

^***^ The gender percentage of all patients in the same register

Table 2 shows the top ten professions in the FWCC register. The majority of the women did kitchen/bakery work, but among the men, jobs varied more widely. In the NBC material, industrial professions (27%) and electricians (23%) dominated.

**Table 2:** Professions of occupational burn patients in Finland 2011–2015

Male		Female			
FWCC		FWCC		NBC*	
Mechanics	729 (22%)	Kitchen/bakery work	2514 (57%)	Industry	7 (27%)
Kitchen/Bakery work	697 (21%)	Waitresses, bartenders	664 (15%)	Electricity	6 (23%)
Excavation	520 (16%)	Sellers	325 (7%)	Property management	5 (19%)
Plumbers	373 (11%)	Nursing	321 (7%)	Road work	4 (15%)
Electrical/phone installation	300 (9%)	Cleaners	217 (5%)	House building	2 (8%)
Welders	286 (9%)	Office and management	173 (4%)	Traffic	1 (4%)
Property management	231 (7%)	Social work	141 (3%)	Waitresses, bartenders	1 (4%)
Lorry drivers	164 (5%)	Teaching	86 (2%)		
Total	3300 (100%)		4441 (100%)		26 (100%)

^*^Genders are not separated since NBC data include only one female (group: waitresses, bartenders)

**Fig. 1:**
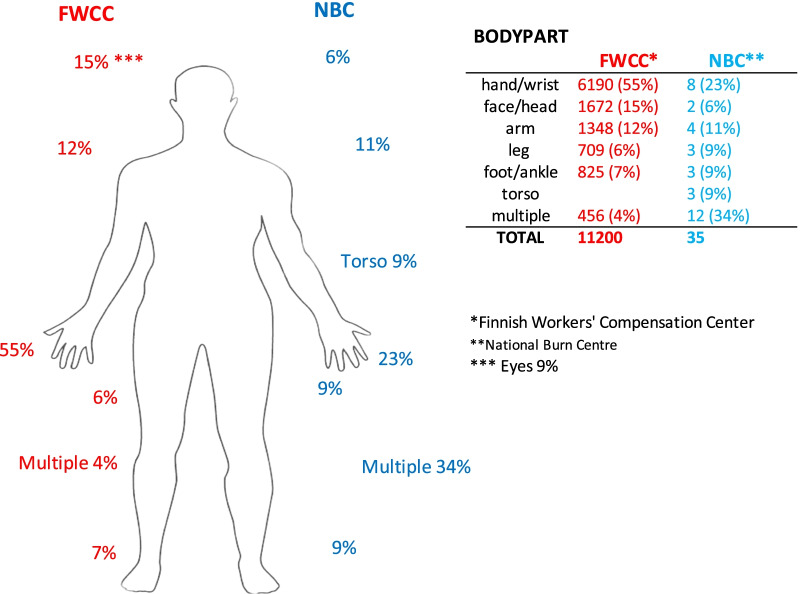
Injured Body Parts of Occupational Burn Patients 2011–2015. *Finnish Workers' Compensation Center. **National Burn Centre. *** Eyes 9%

The injury mechanisms (Table 3) and injured body parts (Fig. 1: Injured body parts of occupational burn patients 2011–2015) differed in the NBC and FWCC registers: In the NBC, the ‘multiple body part’ group dominated, but in the FWCC, hand injuries were the most common.

**Table 3:** Reported injury mechanisms of occupational burn patients in Finland 2011–2015

Mechanism	Male		Female		All	
	FWCC*	NBC**	FWCC	NBC	FWCC	NBC
Heat***	4329 (62%)	5 (20%)	4684 (82%)	0	8923 (71%9	5 (19%)
Dangerous substances (skin/eyes)	1673 (25%)	3 (12%)	754 (13%)	0	2427 (19%)	3 (12%)
Electricity and electrical arc	568 (8%)	15 (60%)	135 (2%)	0	703 (6%)	15 (58%)
Dangerous substances (breathing)	58 (1%)	0	32 (1%)	0	90 (1%)	0
Dangerous substances (swallowed)	7 (0.1%)	0	8 (0.1%)	0	15 (0.1%)	0
Other (hot water, etc.)	247 (4%)	2 (8%)	124 (2%)	1 (100%)	371 (3%)	3 (12%)
Total	6729	25	5737	1	12,529	26

^*^Finnish Workers' Compensation Center (FWCC)

^**^National Burn Center (NBC)

^***^FWCC registry does not specify different mechanisms that result in injuries caused by high temperature. The same burn classification has also been applied to the NBC

Men had more severe burn injuries: In the FWCC register, the sum of compensation represented the severity of the accident. During the study period, insurance companies received 11,234 claims for occupational burn injuries. The mean sum of compensation was EUR 1233 (median EUR 248, SD EUR 8143, max EUR 450,587). Men received 69% of all compensation over EUR 1000.

The mean TBSA of the patients who met the EMSB referral criteria for specialized burn care was 22% (Table 4). The TBSA correlated with length of hospital stay, length of absence from work, and number of follow-up visits. The highest mean TBSA (37%) was in the 51–60- and 61–70-year age groups. The mean age of the patients with a TBSA of 40% or over (n = 7) was 53 years. Their professions were similar to those of the other NBC patients.

**Table 4:** Hospitalization and follow-up data of occupational burn patients’ in NBC of Finland 2011–2015

Age	Number of patients	M/F*	TBSA	LOS**	Follow-up visits	Returned to work
18–30	9	8/1	12	8	6	8
31–40	0	0	0	0	0	0
41–50	8	8/0	18	14	6	4
51–60	6	6/0	37	31	5	4
61–70	3	3/0	37	60	10	1
TOTAL		25/1				17/26
mean of group			22	21	6	
median of group			10	12	3	
range within the group			1–80	2–133	1–18	

^*^Male/female

^**^Length of stay

Recovery among the patients in the NBC was good: 17 patients were able to return to work life (Table 4) and four patients changed professions for different reasons (psychological reasons, inability to stand for long periods). One patient retired (TBSA over 50%) but, based on outpatient reports, had a good quality of life. Two patients with TBSA over 50% died. The FWCC data contained four more deaths, the injury details of which were unknown. These patients probably died during emergency care or in the emergency room.

## Discussion

This study provides information on all occupational burn injuries in Finland in 2011–2015.

According to our knowledge, such comprehensive data on one country has not previously been published.

Our data consisted of two sources which were rather different. The FWCC is a non-medical register that contains literally all the occupational burn cases of a given year. Reporting of any occupational accident is mandatory and based on Finnish legislation. Further employer gains financial benefits by reporting occupational accidents (Tilastokeskus Työtapaturmat 2015). From the NBC, we collected the reports on occupational burn patients who met the EMSB referral criteria for specialized burn care. The NBC admitted during the study period only 26 patients. These cases represented approximately 2/3 of the severe cases in the country that needed tertiary-level burn care. Some other injuries may have been hospitalized in another smaller national burn center till 2015, but still the number of the most severe occupational burn injuries is low.

In Finland, the working-age population was 3,480,000 in 2015 and the unemployment rate was 9.4% (men 9.9%, women 8.8%) (Tilastokeskus 2015; Tilastokeskus 2016). There were a total of 127,000 accidents at work in the same year (62.5% of men), including both accidents at work and commuting accidents. Total number of wage earners in 2015 was 2,090,000 and they had 36,020 accidents at the work place (men 65.2%) that caused absence from work four days or more (Työllisyys ja työttömyys 2015). Absence from work for more than 30 days (average 11 days) was defined as a serious accident and 19.1% of accidents fulfilled this description. The trend is similar for entrepreneurs, but serious accidents were more common among farmers.

Older people had longer absence from work in the same statistics. Burn injuries were rare: 2,0 percent of cases were reported as burn, corrode or frost bite injuries. Based on injury mechanisms heat, electricity or dangerous substances caused 2,7 percent of injuries (Official Statistics Finland 2017).

Several earlier studies have shown that men are at the highest risk of both occupational accidents and burn injuries (Mian et al. 2011; Reichard et al. 2015; Hansen 2019; Hanvold et al. 2019; Tanttula et al. 2018). Our data showed different results: The gender distribution in the FWCC register was almost even (men 54%) but men dominated (25/26 cases) in the NBC data. Based on this result, women tend to have occupational burn injuries almost as often as men, but their injuries are minor. Similarly, men dominated the group in the FWCC, with compensation of over EUR 1000, which supports the result that men sustained more severe occupational burn injuries. Patients also had non-identical professional profiles: The NBC patients mainly worked in industrial professions, electrical injuries being the most common. In the FWCC, the men had similar professions, but the women mainly worked in the service sector, in kitchen and bakery work.

In Finland, women work outside the home almost as often as men, but professions are still segregated. Men dominate in construction work, transportation and logistics, for example, whereas women more often work in the social and service sectors (Terveyden 2022). Risk profiles are very different but one can also speculate whether women have more careful working habits and follow safety regulations more precisely. Another explanation might be that our data covered work-related injuries comprehensively, even minor ones, which earlier studies may have not recognized.

The NBC patients were older than those in the FWCC register. Further, in the NBC, older patients had more severe injuries: In the 51–60- and the 61–70-year age groups, the mean TBSA was 37% vs 22% in the entire NBC data. The mean age in the NBC was 43 years, but those with a TBSA of 40% or over had a mean age of 53 years.

Notably, the NBC data did not contain any 31–40-year-olds, whereas in the FWCC register this age groups was well represented: males 23% and females 20%. We do not know the reason for this. It may be merely a statistical item due to the small number of cases, but other factors may also be at play. This is a group that already has good professional skills but is not overconfident and perhaps follows safety regulations better than older people.

Many earlier studies have shown different results: A US study by Guerin et al. (2020) showed that younger employees suffered more work-related injuries than employees over 24 years of age. The study did not include burn injuries as a separate group but gives nevertheless the overall picture of age and occupational injuries (Guerin et al. 2020). Limitation of this paper is that it includes only employees under 44 years.

Rommel et al. (2016) had similar results in Germany: Older men had less work-related injuries than younger ones (Rommel et al. 2016). In our study, the number of patients in the NBC data was low, but the trend was obvious. Those with major injuries had high-risk professions (industrial, transportation, etc.). The accidents were sudden (transport accidents, falling into hot water, explosions) but in principle, avoidable events. We did not study the working conditions or events prior to the accident. Experienced employee might also be tempted to perform repeated tasks routinely, and some safety habits may become neglected over time. In addition, safety regulations are very strict in Finland and young people may not be allowed to carry out dangerous work before proper training, which may not be the case in many other countries.

Earlier, Finnish studies have shown that TBSA accurately predicts return to work (Palmu et al. 2015; Tanttula et al. 1997). In our study, TBSA was only available in the NBC medical reports and correlated with length of absence from work.

Almost all the patients NBC returned to work: Only one patient retired, and five patients had to change professions but returned to work life. A Danish study by Rikhardsson and Impgaard (2004) showed that the average cost of occupational accident per employee is EUR 4200, 60% of which is due to absence from work, emphasizing that return to work life is also an important goal from the financial point of view (Rikhardsson and Impgaard 2004).

Finnish legislation on occupational injuries is strong, and the employer is responsible for compensation. In the case of retirement due to occupational accidents, compensation is 85% of the employee’s former salary until the employee is 65 years old. Costs are covered by insurance, but to the employer the loss of competent worker is expensive: In the short run, the employer must hire a new employee, who probably lacks the required know-how. Work capacity is lower because of the training period, and in the long run, the prices of insurance may rise due to severe injuries. On the individual level, retirement at a young age is devastating, and highly expensive for society. One important goal of specialized burn care of working-aged people is to guarantee the ability to return to work life. According to the NBC data, this was well achieved.

Eurostat is the statistical office of the European Union, responsible for publishing high-quality, Europe-wide statistics. According to their data, in 2018 the incidence of occupational accidents in Europe was 1659/100,000. In Finland, the corresponding figure was clearly higher, at 1887/100,000. It is notable that even in Eurostat’s own analysis reporting mistakes are recognized (Eurostat 2022). In Finland, everyone has a unique social security number and general attitudes toward registers are positive. Based on Finnish legislation, actors in social and health care are registered and must report patient/client information (Finlex 2015 ; Harala 2010). *Reporting is based on compensation claims which insurance companies have received*. Due to this reporting is separate from employer, who might like to hide occupational accidents. Further, insurance companies are separate from employers and have no reason not to report accidents compensated by them. In a highly educated and organized country with strict safety rules, the number of reported occupational injuries is very close to the true number. It can be postulated that the same does not apply to all the European countries and their statistics, which should be taken into consideration when comparing the figures.

Safety at work has improved substantially over recent decades in Finland, and the number of fatal occupational accidents has declined. The expected retirement age has risen in all age groups (Statistics Finland 2022; Oksa et al. 2019).

Both data sources have limitations: The FWCC register is not a medical register and does not include ICD-10 diagnoses; the data are thus not directly comparable with medical data.

The NBC occupational burn patient data from 2011 to 2015 were collected based on ICD-10 Y96 codes. The data search yielded only 51 cases, of whom only five were women. Of these, 26 patients met the EMSB criteria (one woman). During the same time period, the NBC had approximately 20 patients per year with burn injuries with TBSA of 20% or higher and who needed intensive care. Further statistical analysis of genders or age groups is not possible due to the small number of cases. On the other hand, having such a small number of occupational burn patients in a tertiary-level burn is a desirable outcome.

The FWCC register includes practically all the occupational burn injuries in the entire country during the study period. Unfortunately, the register does not contain information on the length of hospital stay, the number of outpatient visits, etc. These variables form the total cost of burn injury together with many other factors (physio- and occupational therapy, mental care, special clothing, traveling costs, etc.). Furthermore, the FWCC register does not include TBSA. Due to this, the severity of the burn injury is evaluated indirectly, based on the total sum of compensation.

One limitation of our study is that we are unable to provide denominator data concerning Finnish population. Due to this, we cannot compare directly burn cases with the total number of men or women working with certain profession.

## Conclusions

These data on all work-related burn injuries in one country recognize several high-risk groups. Minor burn injuries are common in young adults working in kitchen and bakery work, whereas elderly men working in transports and industry sustain the most severe burn accidents. Although return to previous work was mostly achieved even after severe accidents, elderly employees may have had earlier health problems and been at a higher risk of retirement. Retirement due to occupational accidents is very expensive, and thus, focusing on prevention and safety regulations is important.

Finland has achieved its goal improving safety at work during the last decades, but more detailed cost analysis of occupational burn accidents would be beneficial, especially after centralization of the care of severe burn injuries.

## Data Availability

The datasets generated during and/or analyzed during the current study are not publicly available due to possibility to identify patients but are available from the corresponding author on reasonable request.

## Abbreviations

ANZBA: Australian and New Zealand Burn Association
EMSB: Emergency Management of Severe Burns
ESAW: European Statistics on Accidents at Work
FWCC: Finnish Workers’ Compensation Center
HRQL: Health-related quality of life questionnaire
ICD10: International Classification of Diseases
LOS: Length of stay
NBC: National Burn Center
TBSA: Total body surface area
